# Characterization of Hepatitis C Virus IRES Quasispecies – From the Individual to the Pool

**DOI:** 10.3389/fmicb.2018.00731

**Published:** 2018-04-24

**Authors:** Václav Vopálenský, Anas Khawaja, Luděk Rožnovský, Jakub Mrázek, Tomáš Mašek, Martin Pospíšek

**Affiliations:** ^1^Department of Genetics and Microbiology, Faculty of Science, Charles University, Prague, Czechia; ^2^Clinic of Infectious Medicine, University Hospital Ostrava, Ostrava, Czechia; ^3^Institute of Public Health in Ostrava, Ostrava, Czechia

**Keywords:** HCV, hepatitis C virus, IRES, quasispecies, DsRed2, EGFP, flow cytometry, DGGE

## Abstract

Hepatitis C virus (HCV) is a single-stranded positive-sense RNA virus from the genus *Hepacivirus*. The viral genomic +RNA is 9.6 kb long and contains highly structured 5′ and 3′ untranslated regions (UTRs) and codes for a single large polyprotein, which is co- and post-translationally processed by viral and cellular proteases into at least 11 different polypeptides. Most of the 5′ UTR and an initial part of the polyprotein gene are occupied by an internal ribosome entry site (IRES), which mediates cap-independent translation of the viral proteins and allows the virus to overcome cellular antiviral defense based on the overall reduction of the cap-dependent translation initiation. We reconsidered published results concerning a search for possible correlation between patient response to interferon-based antiviral therapy and accumulation of nucleotide changes within the HCV IRES. However, we were unable to identify any such correlation. Rather than searching for individual mutations, we suggest to focus on determination of individual and collective activities of the HCV IRESs found in patient specimens. We developed a combined, fast, and undemanding approach based on high-throughput cloning of the HCV IRES species to a bicistronic plasmid followed by determination of the HCV IRES activity by flow cytometry. This approach can be adjusted for measurement of the individual HCV IRES activity and for estimation of the aggregate ability of the whole HCV population present in the specimen to synthesize viral proteins. To detect nucleotide variations in the individual IRESs, we used denaturing gradient gel electrophoresis (DGGE) analysis that greatly improved identification and classification of HCV IRES variants in the sample. We suggest that determination of the collective activity of the majority of HCV IRES variants present in one patient specimen in a given time represents possible functional relations among variant sequences within the complex population of viral quasispecies better than bare information about their nucleotide sequences. A similar approach might be used for monitoring of sequence variations in quasispecies populations of other RNA viruses in all cases when changes in primary sequence represent changes in measurable and easily quantifiable phenotypes.

## Introduction

Although the hepatitis C virus (HCV) is an important pathogen that infects between 130 and 170 million people worldwide, the existence of the virus was not demonstrated until 1989 (Choo et al., 1989; Gravitz, 2011). The HCV often induces chronic infections with a long asymptomatic initial phase that can ultimately result in liver cirrhosis and cancer. Approximately 15–20% of patients infected with HCV develop liver cirrhosis, and subsequently, some will develop liver carcinoma. A disease etiology has been reviewed recently in Tang et al. (2016). Many infected patients are unaware of their disease for many years and do not undergo treatment, which increases the probability of serious health complications. The distribution of HCV-infected individuals is very unequal around the world, and the estimates vary from 0.1 to 2% in developed countries to more than 10% in some Asian and African countries (Lavanchy, 2011).

HCV is a single-stranded positive-sense RNA virus from the genus *Hepacivirus*, a member of the *Flaviviridae* family. The viral genomic RNA is 9.6 kb long and codes for a single large protein (more than 3000 amino acids in length; Choo et al., 1991), which is co- and post-translationally cleaved by cellular and viral proteases into mature viral structural and non-structural proteins (for reviews, see Bartenschlager et al., 2013; Li et al., 2015; Bukh, 2016; Douam et al., 2016). Viral RNA is bordered by highly structured 5′ and 3′ untranslated regions (UTRs), which carry essential functions for virus replication, transcription, packaging of viral RNA into virions, and for the initiation of viral polyprotein synthesis (reviewed in, e.g., Dustin et al., 2016). Both UTRs assemble into complicated secondary and tertiary structures and interact with viral and cellular proteins and with each other (Song et al., 2006; Niepmann, 2013). Most of the 5′ UTR and an adjacent part of the core gene are occupied by the HCV internal ribosome entry site (HCV IRES), which can initiate the translation of the viral polyprotein in a cap-independent manner (Wang et al., 1993). The HCV IRES spans a region of ∼341 nucleotides and folds into four structurally distinct domains (Brown et al., 1992). Unlike most other known viral IRESs (Lozano and Martinez-Salas, 2015), the HCV IRES has minimal requirements for cellular translation initiation factors and contains only binding sites for the multimeric translation initiation factor 3 (eIF3; Sizova et al., 1998). The sequence and structural conservation of the HCV IRES are important to maintain its direct and functional contacts with the translational machinery and consequently provide an optimal yield of viral protein synthesis. We recently reviewed the close relationship between the structure and function of the HCV IRES (Khawaja et al., 2015). Various biochemical and structural studies have demonstrated the importance of the conservation and specificity of HCV IRES domains. Whereas domain I is expendable for the activity of the HCV IRES, even single-nucleotide substitutions or indels in domains II–IV may have a severe impact on HCV IRES efficacy in translation initiation. A recent listing of currently known variations in the HCV IRES, including their impact on HCV IRES activity, has been published in the HCV IRES Variation Database^1^ (Floden et al., 2016).

Phylogenetic studies have suggested that there are seven genotypes and almost 70 subtypes of HCVs (Smith et al., 1997; Echeverria et al., 2015). However, the variability generated by the viral RNA polymerase lacking proof-reading activity (Simmonds, 2004) is enormous even within individual subtypes. Whereas there is much variation within the genomic sequence of the HCV, the 5′ UTR containing the IRES is relatively highly conserved among all genotypes (Laporte et al., 2000). In patients, HCV circulates as a population of different but closely related viral variants (Martell et al., 1992), known as quasispecies, that are derived from one master viral genome. Multiple replicating viral quasispecies can be isolated from the same patient, even from the same organ (Cabot et al., 1997; Pawlotsky, 2006; Domingo and Gomez, 2007).

**FIGURE 1:**
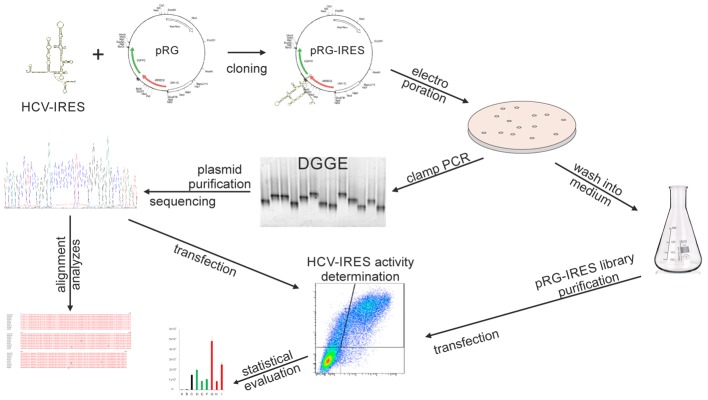
Graphical abstract summarizing the experimental workflow.

The major objective of our work is the development of a methodological approach to study the diversity of individual HCV IRES viral variants occurring in a single patient. We focus on the development of a versatile system suitable for measuring HCV IRES activities at both the level of the single individual IRES sequence and the level covering the entire population of IRES variants in a clinical sample at a given time. A graphical abstract summarizing the experimental workflow is presented in **Figure 1**.

## Materials and Methods

### Patients

Peripheral blood samples from the following three patients were used throughout this study: Patient 4 (P4, male, 22 years, HCV genotype 1a, viral load 1.3E7 IU/ml at the time of RNA purification, medium biochemical activity, non-treated); Patient 7 (P7, female, 40 years, infected by blood transfusion, HCV genotype 1b, viral load 2.0E7 IU/ml at the time of RNA purification, non-responder (NR), prior to sampling for HCV IRES analysis underwent three unsuccessful treatments with interferon alfa-2b [6 months], interferon alfa-2b, and ribavirin [12 months], interferon alfa-2b, ribavirin, and amantadine [12 months]; and Patient 9 (P9, male, 24 years, infected probably by sexual transmission, HCV genotype 1a, viral load 4.9E6 IU/ml at the time of RNA purification, low biochemical activity, non-treated). All subjects gave their informed consent for participation in the study. The study was conducted in accordance with the Declaration of Helsinki, and the protocol was approved by the Ethics Committee of the University Hospital Ostrava (Project No: GA301/07/0607).

### RNA Purification and Reverse Transcription

RNA from patients with HCV was purified using a QIAamp Viral RNA Mini Kit (Qiagen) according to the manufacturer’s protocol. Total RNA was converted to cDNA as follows: 10 μl viral RNA was incubated with 0.15 μg random hexamers (Invitrogen) and 200 U SuperScript III Reverse Transcriptase (Invitrogen) in a 25 μl reaction for 60 min at 50°C, followed by removal of viral RNA using 2 U *Escherichia coli* RNase H and incubation at 37°C for 20 min. Viral cDNA was ethanol precipitated and used for subsequent PCR amplification.

### PCR Amplification of the HCV IRES, Plasmid Construction, and Analysis

The HCV IRES sequence was PCR amplified from viral cDNA with forward (HCVIRESf-SalI) and reverse (HCVIRESr-BamHI) primers containing SalI and BamHI restriction sites, respectively (**Table 1**). PCR fragments were purified using a High Pure PCR Product Purification Kit (Roche) and digested using BamHI and SalI restriction endonucleases. Full-length HCV IRES fragments (nt 1–385) were analyzed using 1.5% agarose electrophoresis, purified from the agarose gel using a GENECLEAN III kit (MP Biomedicals), and cloned into a bicistronic plasmid pRG (comprises DsRed2 and EGFP reporter genes under the control of CMV-IE promoter; Masek et al., 2007), which was linearized using SalI and BamHI and dephosphorylated with shrimp alkaline phosphatase (Fermentas). The ligation mixture was subsequently electroporated into *E. coli* XL1-Blue cells and plated on 2xTY agar plates containing kanamycin (75 μg/ml). Individual monocolonies were subjected to plasmid minipreparation (Holmes and Quigley, 1981), and clones containing full-length HCV IRES fragments were selected after digestion with SalI and BamHI restriction endonucleases. Alternatively, agar plates containing individual monocolonies were washed with 2xTY medium containing kanamycin one day after electroporation. The washed-off cell suspension was inoculated in 50 ml 2xTY medium containing kanamycin (75 μg/ml), grown overnight, and used for plasmid library purification. All plasmids used for transfection of cell cultures were purified using a QIAGEN Plasmid Mini/Midi Kit (Qiagen). All clones were verified by digestion with restriction endonucleases and sequencing using DsRedSeqPrimer (**Table 1**).

**Table 1:** Primers used in this study.

Primer name	Sequence^1^
HCVIRESf-SalI	5′-AAAGTCGACGCCAGCCCCCTGATGGGGGCGACAC-3′
HCVIRESr-BamHI	5′-ACGGATCCGTGTTACGTTTGGTTTTTCTTTGAGGTTTAGG-3′
DsRedSeqPrimer	5′-AGCTGGACATCACCTCCCACAACG-3′
HCVIRESf-Sal-clamp	5′-CGCCCGCCGCGCCCCGCGCCCGTCCCGCCGCCCCCGCCCGGTCG
	ACGCCAGCCCCCTGATGGGGGCGACAC-3′


*^*1*^SalI and BamHI restriction sites are underlined.*

### Cell Cultures, DNA Transfection, and Flow Cytometry Analysis

The human epithelial cell line CCL-13 (also known as Chang cells) was cultured in Dulbecco’s Modified Eagle’s Medium (DMEM; Sigma) supplemented with 100 U/ml penicillin, 100 μg/ml streptomycin, and 10% fetal bovine serum (Gibco) at 37°C under 5% CO_2_ and 95% relative humidity. The cells were detached by a trypsin-EDTA solution (Gibco) and split into 6 cm dishes at a 1:5 ratio. For transient transfections, the cells were plated in 24-well tissue culture plates 24 h before transfection at approximately 40% confluency. The next day, the cells were transfected using 3.6 μl ExGen transfection reagent (Fermentas) mixed with 1 μg plasmid DNA per well. Forty-eight hours after transfection, the cells were collected by trypsinization and resuspended in DMEM medium. Samples were analyzed by flow cytometry using a BD LSRII device and a Coherent Sapphire 488-20 DPSS laser to excite the cells at 488 nm, a 530/30 nm band path filter to detect EGFP, and a 585/42 nm band path filter to detect DsRed2. At least 50,000 living cells were counted. Depending on transfection efficiency, this corresponds to approximately 20,000 DsRed2-positive cells that were used for statistical calculations. Flow cytometry results were analyzed using FlowJo software (Tree Star).

### Colony Clamp PCR

Bacterial monocolonies were selected from agar plates after growing overnight and transferred into 300 μl ddH_2_O in Eppendorf tubes. The tubes were vortexed and incubated at 96°C for 10 min, cooled down to room temperature for 5 min, heated again at 96°C for 10 min, and rapidly spun down. The supernatant was used as a template in the PCR reaction with GC clamp primers for further usage of PCR product in DGGE analyses. The HCV IRES fragment containing the clamp sequence at its 5′ end was amplified either directly from the heated bacterial cells (see above) or with purified plasmid DNA as a template. PCR was performed using HCVIRESf-Sal-clamp and HCVIRESr-BamHI (**Table 1**) primers as follows: 3 min at 95°C, 25 cycles of 30 s at 94°C, 30 s at 56°C, 45 s at 72°C, and 10 min at 72°C.

### Denaturing Gradient Gel Electrophoresis

DGGE was performed following the manufacturer’s instructions (INGENYphorU-2) using a 6% separating polyacrylamide gel (30% acrylamide/0.8% bisacrylamide). To prepare the 6% polyacrylamide gel containing a 55–70% gradient of the denaturing agent, the following two solutions were mixed: the first contained 55% denaturant (4.6 ml 30% acrylamide/0.8% bisacrylamide; 5.32 g 7 M urea; 5.06 ml 40% formamide in a total volume of 23 ml), and the second contained 70% denaturant (4.6 ml 30% acrylamide/0.8% bisacrylamide, 6.77 g 7 M urea; 6.44 ml 40% formamide in a total volume of 23 ml). Sixty-eight microliters of ammonium persulfate (10%) and 31.5 μl of TEMED were added into both solutions to induce gel polymerization. The gel was casted in between a glass plate sandwich using gradient maker according to the manufacturer’s instructions. After 20–30 min of polymerization, the stacking gel, which comprised 2 ml acrylamide solution (30% acrylamide/0.8% bisacrylamide), 0.1 ml 0.5X TAE buffer, 7.9 ml ddH2O, 31.5 μl ammonium persulfate (10%), and 19 μl TEMED, was poured. Combs were placed in between the glass plates, and the gel was allowed to polymerize for at least 2 h. The cassette was placed in a buffer tank filled with 0.5x TAE, and the gel was run at 100 V for 17 h at 60°C. The gel was stained for 10 min with ethidium bromide (final concentration 0.175 μg/ml) and further washed with water for another 10 min before images were acquired.

### Statistical Analysis

Results of independent flow cytometry measurements were evaluated as mean EGFP fluorescence intensity per cell that was calculated from approximately 20,000 measurements taken from individual DsRed2-positive cells in the sample. For the comparison of HCV IRES activities measured by flow cytometry, we performed at least seven biological replicates, and each was represented by independent pRG-IRES and/or pRG-IRES-library transfection. The significance of the differences between the activities of different HCV IRESs was tested using one-way ANOVA followed by *post hoc* Tukey’s HSD test and further confirmed with Scheffe multiple comparison. The normal distribution of the data was confirmed by Shapiro–Wilk test.

## Results and Discussion

### Mapping Nucleotide Variations in the HCV IRES From Sustained Responders and Non-responders to Interferon Treatment – A Bioinformatical Approach

Over the past few years, the treatment of HCV infection has significantly progressed with the introduction of various classes of direct-acting antivirals (DAAs) targeting viral NS3/4A protease, NS5A protein, and/or NS5B RNA polymerase, which have greatly improved sustained viral response (SVR) rates in treated patients. However, the resistance to DAAs has appeared almost concurrently with their introduction to clinical practice, and their efficiency depends on the genotype/subtype and the fitness of the resistant viral populations (Chen et al., 2016; Jimenez-Perez et al., 2016; Sarrazin, 2016). These findings together with the prohibitively high cost of most new DAAs and slow legislative processes in many countries are the reasons why the standard therapy for hepatitis C, which is based on PEGylated interferon and a nucleoside analog, ribavirin, is still broadly used (Walsh et al., 2015). Currently, interferon-based therapy is still the only approved treatment for pediatric patients (Ohmer and Honegger, 2016).

The HCV IRES is essential for the translation of the viral proteins, and therefore, it is not surprising that more studies have aimed to investigate the possible correlation between patient response to antiviral therapy with interferon and mutations in the HCV IRES. However, the obtained results are rather contradictory. Some studies could not find any significant correlation between interferon-α treatment and genetic changes within the HCV IRES, accumulation of specific mutations in IRES, and/or IRES activity. Similarly, no specific differences have been observed in the activity and/or sequence variations of the HCV IRESs between sustained responders (SRs) and non-responders (NRs) to interferon-based antiviral therapy (Yamamoto et al., 1997; Saiz et al., 1999; Soler et al., 2002; Thelu et al., 2004). Conversely, other authors have described the selection of new HCV IRES variants in SRs but not in NRs (Lu et al., 1999), differences in the distribution of mutations within domain III between SRs and NRs (El Awady et al., 2009), shift in accumulating mutations from domain III to domain II in NRs during interferon–ribavirin therapy (Ogata et al., 2008), differences in the distribution and frequency of single-nucleotide changes within the IRES between SRs and NRs during the first days of interferon treatment (Thelu et al., 2007) and less single-nucleotide changes within the HCV IRES among NRs when compared with those among SRs, and the low relative translation efficiency of HCV IRES elements obtained from SRs compared with the IRESs obtained from NRs (Yasmeen et al., 2006).

**FIGURE 2:**
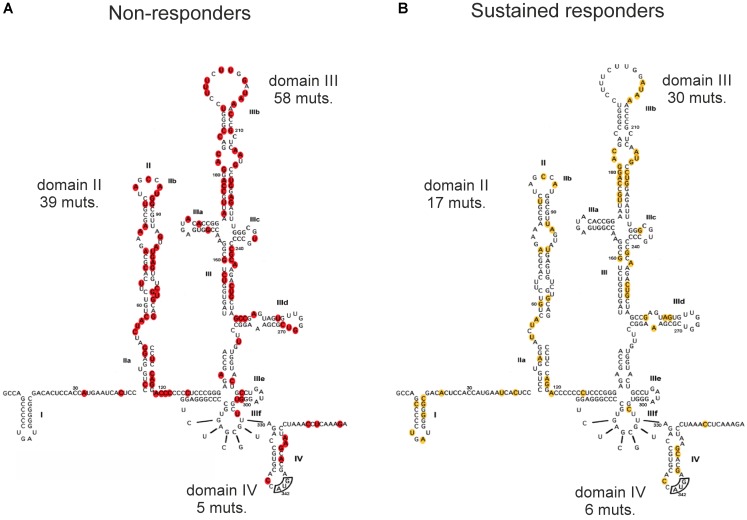
Distribution of mutations across the HCV IRES from patient samples with respect to sustained response and no antiviral response to HCV treatment. **(A)** Sustained responders (SRs), localization of 53 individual mutations found in the literature. **(B)** Non-responders (NRs), localization of 102 individual mutations found in the literature (Yamamoto et al., 1997; Lu et al., 1999; Saiz et al., 1999; Soler et al., 2002; Thelu et al., 2004; Yasmeen et al., 2006; Ogata et al., 2008; El Awady et al., 2009). Total numbers of mutations (muts.) found within the corresponding domains in the SR and NR groups are depicted.

We decided to analyze nucleotide changes in the HCV IRESs obtained from SRs and NRs to the standard combination therapy of IFNα and ribavirin, which were published in the studies mentioned above (Yamamoto et al., 1997; Lu et al., 1999; Saiz et al., 1999; Soler et al., 2002; Thelu et al., 2004; Yasmeen et al., 2006; Ogata et al., 2008; El Awady et al., 2009). In these studies, the HCV IRES cDNA samples were obtained from patients’ blood specimens that were collected before or after the therapy and sequenced. The objective was to search for specific sequences or regions that might be involved in inducing a sustained antiviral response or no response in patients by analyzing the location of nucleotide variations in the HCV IRES in both patient groups. The number of patients included from all these studies was 110, from which 61 patients showed sustained responses and the remaining 49 were NRs. We mapped all the published variations from these studies to the HCV IRES secondary structure to understand the possible correlation that may exist between the occurrence of mutations in different HCV IRES domains and the patient response to therapy. Even though this approach gave us an advantage to evaluate a large cohort of 110 patients, we did not detect any notable pattern and/or association between therapeutic responsiveness and the mutation occurrences in HCV IRES domains and subdomains as it is seen from the percentage of the mutated nucleotides (calculated from the total number of nucleotide variations found in HCV IRESs of the respective group of patients) occurring in different HCV IRES domains in SRs and NRs (**Table 2**) and can be visually inspected in **Figure 2**, where positions of all the mutated nucleotides were highlighted. The presence of mutations appears to be rather random in both the SRs and NRs, and no direct link could be observed or developed for the location of variations in the HCV IRES and their possible effectiveness in inducing a sustained or no antiviral response. Taking into consideration that authors of different studies included in this evaluation used different approaches and some of them did not focus on the frequency of the occurrence of published nucleotide variations, registration of the occurrence of nucleotide variations at the specific HCV IRES positions was the only method available (**Figure 2**). If we expect a rather random occurrence of mutations along the HCV IRES, we can calculate the normalized occurrence of mutations, expressed as a relative occurrence of mutations per nucleotide. We performed such a calculation separately for structurally and functionally distinct HCV IRES domains II–IV from both SRs and NRs. Statistical evaluation using Welch’s *t*-test did not show any significant difference in a mean value of the normalized occurrence of mutations in HCV IRES domains between SRs and NRs (*P* = 0.61, Supplementary Table S1). The overall mutation occurrence in the HCV IRES was slightly higher in NRs (≈2.08 mutations/patient) than that in SRs (≈0.87 mutations/patient). However, some of these studies did not focus on the frequency of the mutation occurrence, and these numbers might be biased. We did not aim to evaluate a possible impact of nucleotide variations collected from these studies (Yamamoto et al., 1997; Lu et al., 1999; Saiz et al., 1999; Soler et al., 2002; Thelu et al., 2004; Yasmeen et al., 2006; Ogata et al., 2008; El Awady et al., 2009) to the HCV IRES structure and function. Such an evaluation can be complicated by the presence of multiple mutations in the single IRES and their possible long-range inter- and intra-domain functional interactions (Khawaja et al., 2015). The HCV IRES variations used in this analysis, along with their activities in translation, whenever were available, are publicly accessible in the HCV IRES Variation Database at http://hcvivdb.org (Floden et al., 2016).

**Table 2:** Distribution of mutations within the HCV IRESs isolated from sustained responders (SRs) and non-responders (NRs) to interferon/ribavirin therapy.

Sustained responders (SRs)			Non-responders (NRs)		
	
Domain	Number of mutations	Occurrence (% total mutations)	Domain	Number of mutations	Occurrence (% total mutations)
Domain II	17	32%	Domain II	39	38%
Domain III	30	57%	Domain III	58	57%
Domain IV	6	11%	Domain IV	5	5%
Total	53			102	


*The numbers in the table summarize the number of mutations across the HCV IRES domains found in the literature for SRs and NRs. Absolute numbers of identified mutations within the respective domain are shown, as well as their abundance (in %) relative to the total number of mutations in the corresponding patient group. This table is related to **Figure 2**.*

### Preparation of HCV IRES Libraries From Clinical Samples

The contradictory results obtained by different studies with respect to nucleotide and functional variability in the HCV IRES in SRs and NRs may reflect the different approaches used by these studies and also, to some extent, their slightly different aims. We wanted to develop and test a universal approach that would allow for efficient and cost-effective characterization of the pool of the HCV IRESs occurring within one patient sample. As demonstrated by previous studies (Yamamoto et al., 1997; Lu et al., 1999; Saiz et al., 1999; Soler et al., 2002; Thelu et al., 2004; Yasmeen et al., 2006; Thelu et al., 2007; Ogata et al., 2008; El Awady et al., 2009) and our abovementioned analysis, picking and analyzing individual sequences from the vast amount of viral quasispecies present in a sample do not lead to conclusive results. Clearly, the most important feature of the HCV IRES is its control of the synthesis of the viral polyprotein. We wanted to monitor HCV IRES activity both at the individual and collective level and to directly proceed to the sequence analysis of the selected individual clones. To fulfill this task, we decided to use a pRG bicistronic plasmid that we previously developed for the analysis of IRESs and cryptic transcription. The system is based on the transient production of bicistronic mRNA coding for the fluorescent proteins DsRed2 and EGFP in CCL-13 or Huh7 cells and possesses very low intrinsic cryptic transcription and cryptic splicing activities. These features are indispensable for sensitive and reliable analysis of IRES activity (Vopalensky et al., 2008). The pRG plasmid was used to prepare representative HCV IRES libraries from the patient samples tested. The obtained libraries were subsequently used for the functional and sequence analyses of the individual HCV IRES clones or whole libraries.

To test our approach, we purified total RNA from three clinical samples provided by three patients chronically infected with HCV genotype 1. Two samples (P4 and P9) were obtained from two young men before their treatment. Another sample (P7) was obtained from a 40-year-old woman who underwent three unsuccessful treatments with interferon alfa-2b alone, interferon alfa-2b in combination with ribavirin, and finally with interferon alfa-2b in combination with ribavirin and amantadine. The total RNA purified from each sample was reverse-transcribed to cDNA and amplified using HCV IRES-specific primers. The libraries of HCV IRES amplicons (nt 1-385) originating from the individual patients and representing the IRES variability in selected patients at a given time were cloned into the pRG plasmid (Vopalensky et al., 2008). Maximal ligation efficiency, a low number of empty plasmids (lacking the HCV IRES), and oriented insertion were obtained using sticky ends cloning on both sides and dephosphorylation of the digested pRG plasmid. This approach led to very high (≈98%) efficiency of the ligation reaction, as detected using restriction endonuclease mapping of the individual clones obtained after electroporation into bacterial cells. In other words, out of every 100 bacterial colonies analyzed, only ≈2 did not carry the recombinant plasmid containing the HCV IRES. The yield was hundreds to thousands of colonies for each pRG-IRES cDNA library. The obtained libraries were subsequently further processed by three different ways. First, pRG-IRES clones were randomly selected and analyzed, and those containing full-length HCV IRES cDNA were used either for the determination of IRES variants by denaturing gradient gel electrophoresis (DGGE) or were pooled together to create a sublibrary of the preselected clones (further referred to as *sc*). The remaining bacterial colonies of the particular library were washed off the primary plate, collected, and used for plasmid preparation as a library (further referred to as a *wash*), representing the largest and most complete spectrum of the HCV IRESs in the source blood specimen.

### Analysis of Individual Clones From the HCV IRES Libraries

**FIGURE 3:**
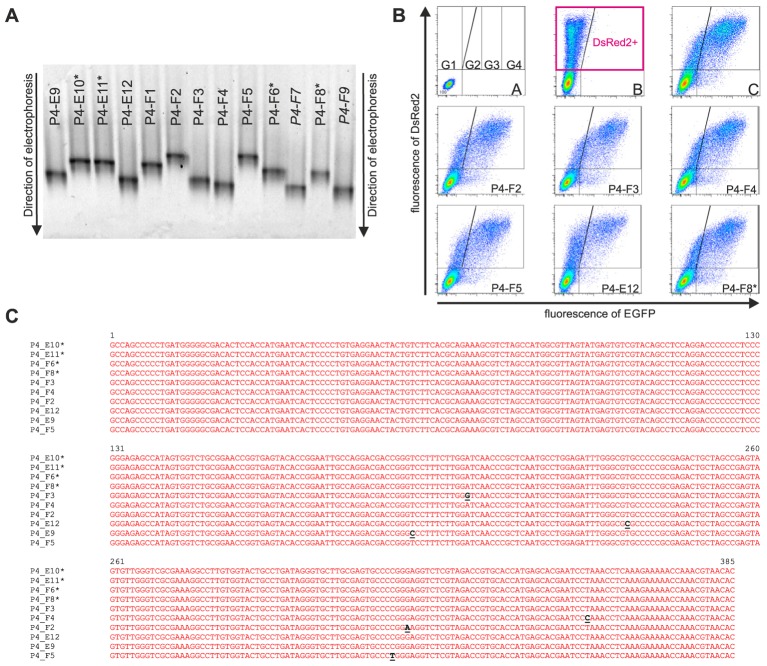
Results documenting a workflow of the analysis of individual HCV IRES clones. Different stages of analysis of some clones derived from Patient 4 (P4) are depicted as follows. **(A)** Denaturing gradient gel electrophoresis of selected samples obtained from P4. Clones marked with asterisks (^∗^) showed identical sequences and similar migration pattern in DGGE with a majority of the clones analyzed from the P4 (wild type). Clones shown in italics were not sequenced. The remaining clones were sequenced, and some were analyzed by flow cytometry. **(B)** Flow cytometry analyses of IRES activity of selected HCV IRES clones in a bicistronic pRG system transfected into CCL-13 cells; *Y* and *X* axes represent red (DsRed2) and green (EGFP) fluorescence, respectively. Lines inside the dot plots indicate gating of the corresponding EGFP and DsRed2 cell populations. B/A represents non-transfected cells. B/B represents a flow cytometry analysis of cells expressing only DsRed2 from empty pRG plasmid and serving as a negative control to set up a baseline of the experiment. B/C represents a positive control cells expressing pRG-refIRES bicistronic plasmid containing standard HCV IRES (subtype 1a, from nt 1 to nt 385 of the original sequence; GI:329737). **(C)** Nucleotide sequences of cDNA from selected HCV IRES clones. Mutations found in individual samples are shown in black and underlined. ^∗^Wild-type sequences for a given patient. Complete data are provided in Supplementary Table S2.

**FIGURE 4:**
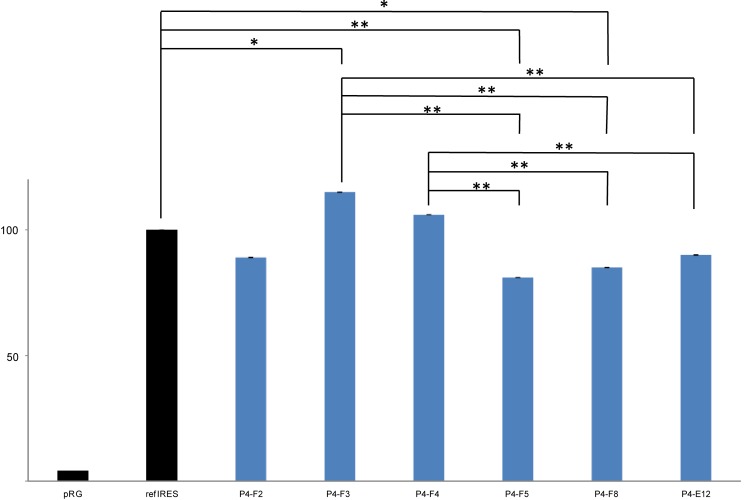
Analysis of HCV IRES activity of selected pRG-IRES clones in human CCL-13 cells using flow cytometry. Columns represent mean EGFP fluorescence intensity per cell, which was calculated from all CCL-13 cells transfected with the respective pRG-IRES, in other words, from all cells expressing DsRed2 above the threshold level in the sample. All the values were normalized to refIRES activity, which was set to 100%. refIRES is the standard HCV IRES (subtype 1a, from nt 1 to nt 385 of the original sequence; GI:329737) cloned into the pRG plasmid (Vopalensky et al., 2008). Empty pRG plasmid serves as a negative control and represents the baseline for the analysis. HCV IRES samples from Patient 4 (P4) are the same as those depicted in **Figure 3** and **Table 3**. At least seven independent biological replicates (independent pRG-IRES transfections) for each of the pRG-IRES plasmids were measured. Significant differences are marked with double asterisks (*p* ≤ 0.01) or an asterisk (*p* ≤ 0.05).

Approximately 150 randomly picked bacterial colonies containing the full-length HCV IRES clones inserted into the pRG plasmid were selected from each library. All clones were individually stored in separate wells of 96-well plates. Sixty-three to 70 clones from each library obtained from samples P4, P7, and P9 were subjected to DGGE analysis, which allowed rapid determination of mutant clones. Briefly, the IRES cDNAs from individual clones were amplified using a colony clamp PCR technique. Obtained amplified DNA was separated by DGGE electrophoresis, and the individual clones were classified by the electrophoretic mobilities of the obtained amplicons. Because all the clones were selected to contain the full-length HCV IRES cDNA, the differences in electrophoretic mobilities of GC-clamped amplicons reflected only variations in the nucleotide sequences of the respective HCV IRESs. Representatives of all the electrophoretic mobility classes were sequenced, and their IRES activities were measured by flow cytometry after direct transfection of the pRG-IRES plasmids into CCL-13 human epithelial cells or Huh7 cells derived from a human hepatocellular carcinoma. An example of this analysis is shown in **Figure 3** and **Table 3**. The pRG-IRES plasmid comprises two reporter genes, DsRed2 and EGFP, which are separated by the analyzed HCV IRES region. Expression of this bicistronic reporter cassette is driven by the human cytomegalovirus immediate-early promoter. The first cistron of the bicistronic mRNA, DsRed2, is translated in a cap-dependent manner, while the second cistron, EGFP, is translated cap-independently under the control of the tested HCV IRES. For each analysis of the HCV IRES activity in pRG plasmid, we used non-transfected cells (**Figure 3B/A**), cells transfected with empty pRG plasmid (**Figure 3B/B**), and cells transfected with pRG-refIRES plasmid containing the reference HCV IRES (**Figure 3B/C**) as standards for setting up the measurement. Only cells transfected with pRG plasmid, which were identified by the presence of DsRed2 (**Figure 3B/B**), were used for further analyses. For fast evaluation of every measurement of the particular HCV IRES activity, we counted the number of cells in each gate from G1 to G4. Note that the G1 gate corresponds mostly to DsRed2 fluorescence, whereas the signal in the G2–G4 gates reflects the increasing green fluorescence of EGFP, which corresponds to the increasing activity of the HCV IRES in particular cells. Examples of this evaluation are shown in **Table 3**. To statistically evaluate the obtained data, we calculated mean EGFP fluorescence intensity per cell, which was calculated from all cells transfected with pRG-IRES, in other words, from all cells expressing DsRed2 above the threshold level in the sample (**Figure 3B/B**). Usually, we prepared and measured seven independent biological replicates (independent pRG-IRES transfections) for each pRG-IRES plasmid by flow cytometry. An example of this analysis is depicted in **Figure 4**.

**Table 3:** An example of the flow cytometry analysis of the activity of HCV IRES clones in a pRG plasmid from Patient 4.

Insert in the pRG plasmid	% of DsRed2-positive cells in the corresponding gated area			
	
	Gate G1	Gate G2	Gate G3	Gate G4
refIRES (1a)	11,5	23,5	20,7	44,3
Empty pRG	84,9	14,9	0,3	0
P4-F2	16,1	18,4	23,4	41,9
P4-F3	12,2	18,6	24,8	44,23
P4-F4	13,3	19,3	23,6	43,8
P4-F5	16,7	19,2	22,6	41,5
P4-E12	30,8	19,8	17	32,3
P4-F8^∗^	15,3	19,1	23,3	46,2


*^∗^The most common sequence according to DGGE mobility, which is called the *wild-type* sequence in this article. The numbers in the table indicate the percentage of DsRed2-positive cells in the corresponding gated area in a selected single experiment. refIRES is the standard HCV IRES (subtype 1a, from nt 1 to nt 385 of the original sequence; GI:329737) cloned into the pRG plasmid. Empty pRG contains no HCV IRES. This table is related to **Figure 3**.*

This analysis allowed us to estimate the diversity of the HCV IRESs in one patient sample. The electrophoretic mobilities in DGGE and sequence variations of the HCV IRES clones were compared with the most common sequence variant in each patient’s specimen, which is also referred to as the wild type. **Figures 3**, **4** and **Table 3** exemplify the whole analysis workflow using the same set of the HCV IRES clones obtained from sample P4. The wild-type clones from sample P4 are marked with asterisk in **Figure 3**. **Figure 3B** shows flow cytometry measurements of HCV IRES activity of the individual clones from sample P4 inserted into the pRG bicistronic reporter plasmid. **Figure 4** summarizes IRES activity of seven independent biological replicates (independent pRG-IRES transfections) of the HCV-IRES clones depicted in **Figure 3**. The empty pRG plasmid (**Figure 3B/C** and **Figure 4/pRG**) serves as a negative control to set up baseline of all the measurements. The pRG-refIRES plasmid bears a reference HCV IRES (GI: 329737) and serves as a positive control (**Figure 3B/C**, **Figure 4/refIRES**, and **Table 3**). The HCV IRES clones with higher and lower activity in comparison to the reference HCV IRES were detected in sample P4. In sample P4, we found 29 IRES clones that exhibited different electrophoretic mobilities from those of the wild type of the 70 analyzed clones; 16 clones carried unique nucleotide sequence variations. In samples P7 and P9, we found 21 and 22 clones with different electrophoretic mobilities of 63 and 69 analyzed clones, respectively. Similar to P4, P7 and P9 revealed 13 and 14 clones with unique nucleotide sequence variations, respectively. In total, we found 43 HCV IRES sequences that differ from the respective wild types in all three patients, which constitute 22.9% of all clones analyzed from P4, 20.6% of all clones from P7, and 20.3% of all clones from P9. Mutant HCV IRESs often contained several nucleotide changes within the IRES sequence. Even though we found that the HCV IRES clones displayed activities distinct from those of the wild type (**Figure 4**), we could not associate these variations with the health status of any of the patients. Despite our small patient cohort, we supported the conclusions of our analysis based on published data from 110 patients as demonstrated above (see section “Mapping Nucleotide Variations in the HCV IRES From Sustained Responders and Non-responders to Interferon Treatment – A Bioinformatical Approach”).

### The Pooled Analysis of HCV IRES Activities

**FIGURE 5:**
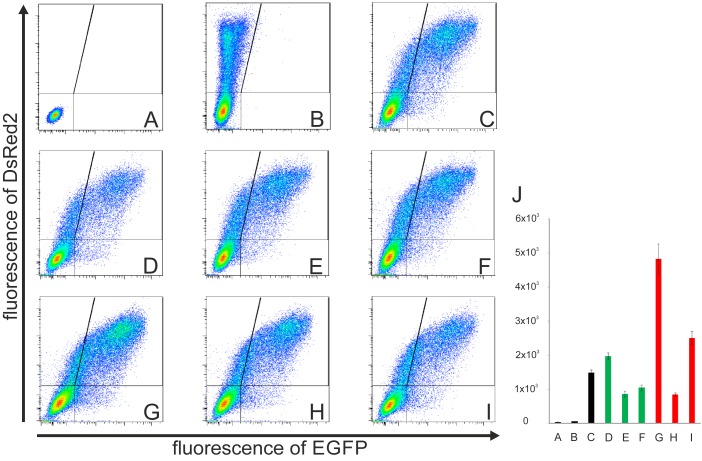
Flow cytometry analyses of the collective activities of HCV IRES libraries transfected into CCL-13 cells. **(A)** Untransfected CCL-13 cells only. **(B)** Cells transfected with the empty pRG plasmid. **(A,B)** These experiments were used to set up gates for the subsequent analysis as depicted by vertical and horizontal lines. **(C)** Cells transfected with the pRG plasmid containing the reference HCV IRES (subtype 1a, from nt 1 to nt 385 of the original sequence; GI:329737). **(D)** Patient 4 – cells transfected with pRG-HCV IRES *sc* library. **(E)** Patient 7 – cells transfected with pRG-HCV IRES *sc* library. **(F)** Patient 9 – cells transfected with pRG-HCV IRES *sc* library. **(G)** Patient 4 – cells transfected with pRG-HCV IRES *wash* library. **(H)** Patient 7 – cells transfected with pRG-HCV IRES *wash* library. **(I)** Patient 9 – cells transfected with pRG-HCV-IRES *wash* library. *Y* and *X* axes represent red and green fluorescence, respectively. Lines inside the dot plots indicate gating of the corresponding EGFP and DsRed2 cell populations. **(D–F)** Each sample consists of approximately 150 positive colonies. **(J)** Seven independent biological replicates (independent transfections) of the samples depicted in **A**–**I**. All measurements differ significantly from all other measurements (*p* < 0.01), except pairs **E** vs. **F**, **E** vs. **H**, and **F** vs. **H**. Complete data are provided in Supplementary Table S2.

As an alternative approach, we pooled all the preselected full-length clones and analyzed the activity of these *sc* libraries as a whole by flow cytometry measurement of their collective IRES activity after transfection of the libraries into CCL-13 or Huh7 cells. The same amount of plasmid/library DNA was used in each experiment. The transfection efficiency varied between 30% and 45%, which did not substantively influence the results because only pRG transfected (DsRed2-positive) cells were evaluated during the procedure, similar to the determination of activities of individual HCV IRES clones (**Figures 3**, **4**). Likewise, we performed the same analysis using the *wash* libraries. The results of the series of pRG transfections and corresponding flow cytometry measurements in one independent experiment with control vectors and either *sc* or *wash* libraries from P4, P7, and P9 blood specimens are shown in **Figures 5A–I**. Differences in the collective activities of the HCV IRESs present in each library are clearly visible even directly from the dot plots referring to each individual pRG-IRES-library transfection and subsequent flow cytometry measurements. Similarly as **Figure 3B** illustrates determination of activity of the individual HCV IRES clones and related **Figure 4** summarizes statistical evaluations of seven such independent analyses, **Figures 5D–I** show flow cytometry measurements of collective IRES activities of the individual pRG-IRES libraries and the related **Figure 5J** shows a statistical evaluation of seven biological replicates (independent transfections) of each pRG-IRES-library and/or the control plasmids. Differences in collective IRES activities between the libraries and the controls as well as most of the differences between the libraries within the same group (*sc* and *wash*) were statistically significant (**Figure 5J**). The results show similar trends for both the pRG-IRES-*sc* (green columns) and pRG-IRES-*wash* (red columns) libraries. Black bars in **Figure 5J** represent the same negative and positive controls as in **Figure 4**. Use of the same positive reference HCV IRES for analysis of the individual IRES clones (**Figures 3**, **4** and **Table 3**) and the HCV IRES libraries (**Figure 5**) allows direct comparison of the IRES activities between the individual clones and libraries prepared from the same patient sample. A bar graph presenting all these analyses together is a part of the supplementary information (Supplementary Figure S1). Activities of the *sc* libraries show similar trends as of the *wash* libraries; however, activities of the P4 and P9 *wash* libraries are much higher. We speculate that these results might be caused by shorter, defective IRESs with a very high activity in the bicistronic pRG system that might be present in viruses from samples P4 and P9. The preselected *sc* libraries, containing only the HCV IRES clones of the correct lengths, display collective activities more comparable to the activity of the reference HCV IRES (**Figure 5J/C,D–F**). We mainly performed these experiments with the aim to develop a new method suitable for global analysis of the collective activities of the majority of HCV IRES variants present in one patient specimen in a given time. We hypothesize that the collective HCV IRES activity calculated from all DsRed2-positive cells and expressed as a mean of EGFP fluorescence intensity per cell closely corresponds to both the activities of individual HCV IRES quasispecies in the sample and to their mutual relative representation. However, these mutual relationships between IRESs in a single patient sample (library) are not simple. The collective activity of the whole library can be inferred neither from the activity of the prevalent HCV IRES variant in a specimen nor from any combination of the individual IRES measurements. Even though the cohort of patients used for the method development was small, our results suggest that the collective activity of all HCV IRES species is lower in a blood sample from NR (P7*sc* and P7*wash*) than that in the samples obtained from non-treated patients (P4 and P9). One potential explanation is that several rounds of unsuccessful treatment might develop a selective pressure, which could lead to a higher fraction of defective and/or less productive viral quasispecies. Another possibility is that the low collective activity of HCV IRES quasispecies in the sample might have a predictive value in terms of patient responsiveness to interferon/ribavirin treatment. However, more experiments need to be performed to answer these questions. Similar results can be obtained using Huh7 cells after the pRG-IRES library transfection.

We show here that analysis of nucleotide variability in the otherwise conserved IRES region of the HCV does not provide any clue to differences of the virus sensitivity to the antiviral treatment. We do not think that usage of more powerful techniques of mass parallel sequencing would substantially help in this or similar tasks. The reason for that is an enormous variability of the virus, large viral populations in the patients, and frequent presence of multiple nucleotide changes within the studied region of the viral genome. Even though the HCV IRES is probably the most studied IRES, we are still not able to predict a possible impact of the nucleotide variations on the HCV IRES activity in many cases. Such a prediction becomes even more complicated in case of multiple nucleotide variations, of which mutual contributions to the IRES activity can be additive or compensatory and resemble gene epistatic networks (Khawaja et al., 2015; Floden et al., 2016). Therefore, we propose to investigate the collective activity of the HCV IRES pool existing in a sample that should directly correspond to the potential of the tested HCV population to synthesize its viral proteins. We hope that our study suggests a direction and a meaningfulness of switching from analysis of individual HCVs in the patient to a broad collective phenotypic analysis of all the viruses present in the patient sample that could more reflect the mutual interaction between the patient and his/her virus population at the given time and under given circumstances. We suggest that a similar approach can be used for monitoring of sequence variations in a quasispecies population of other RNA viruses in all cases when changes in primary sequence represent changes in measurable and easily quantifiable phenotypes.

## Author Contributions

MP and VV conceived and designed the experiments. VV, AK, JM, LR, and TM performed the experiments. VV, MP, and AK analyzed the data and wrote the paper.

## Conflict of Interest Statement

The authors declare that the research was conducted in the absence of any commercial or financial relationships that could be construed as a potential conflict of interest. The reviewer JG and the handling Editor declared their shared affiliation.

## Footnotes

1 http://hcvivdb.org/
