# Supplementary material for: Characterization of Hepatitis C Virus IRES Quasispecies – From the Individual to the Pool
Source: Front Microbiol. 2018 Apr 24;9:731. doi: 10.3389/fmicb.2018.00731 (PMC5928756; doi:10.3389/fmicb.2018.00731)

Supplementary information

Table S1 - Normalized occurrence of nucleotide variations in HCV IRES (expressed as relative occurrence of mutations / nucleotide) was calculated for domain II, III and IV in HCV IRESs obtained from SR and NR. Comparison of IRESs from both groups of patients using these numbers did not reveal a significant difference in occurrence of nucleotide variations.

		relative occurrence of mutations / nucleotide		
		SR	NR	
Domain II		0.004324	0.005135	Welch's *t*-test
Domain III		0.002893	0.002893	*P* = 0.6106
Domain IV		0.004783	0.002174	t = 0.5666
	Mean	0.004000	0.003401	
	SD	0.000986	0.001544	

Table S2 - Independent biological replicates measured by flow cytometry in human CCL-13 cells. Numbers highlighted with the grey background represent mean EGFP fluorescence intensity per DsRed2-positive cell for seven independent biological replicates. * the most common sequence variant for patient #4.

	Mean EGFP fluorescence intensity per DsRed2-positive cell									
Insert in the pRG plasmid	replicate 1	replicate 2	replicate 3	replicate 4	replicate 5	replicate 6	replicate 7	Mean EGFP fluorescence intensity calculated from individual replicates	Standart Deviation	Related to Figure
untransfected CCL-13	32,1	36,5	34,7	38,3	39,2	30,9	33,8	35,071	3,096	3B (panel A); 5A; 5J (bar A)
Empty pRG	46,6	48,6	49,6	48,4	43,3	44,1	50,1	47,243	2,669	3B (panel B); 4; 5B; 5J (bar B)
refIRES (1a)	1520,82	1437,66	1480,5	1416,24	1501,92	1475,46	1520	1478,943	40,037	3B (panel C); 4; 5C; 5J (bar C)
patient #4 - clone P4-F2	1154,4	1150,8	1162,8	1221,6	1452	1267,2	1330,8	1248,514	111,806	3B; 4
patient #4 - clone P4-F3	1694,4	1952,4	1587,6	1432,8	1635,6	1448,4	1586,4	1619,657	174,612	3B; 4
patient #4 - clone P4-F4	1592,4	1610,4	1596	1470	1321,2	1360,8	1470	1488,686	117,041	3B; 4
patient #4 - clone P4-F5	1154,4	1093,2	1161,6	1062	1111,2	1243,2	1189,2	1144,971	61,476	3B; 4
patient #4 - clone P4-F8*	1154,4	1111,2	1281,6	1305,6	1273,2	1232,4	1074	1204,629	91,204	3B; 4
patient #4 - clone P4-E12	1334,4	1200	1430,4	1274,4	1410	1203,6	1034,4	1269,600	138,028	3B; 4
patient #4; pRG-HCV IRES *sc* library	1925	2037	1924	1799	2096	2038	1961	1968,571	98,690	5D; 5J (bar D)
patient #7; pRG-HCV IRES *sc* library	871	903	976	755	800	761	931	856,714	86,427	5E; 5J (bar E)
patient #9; pRG-HCV IRES *sc* library	1068	1141	1133	925	975	1047	989	1039,714	81,377	5F; 5J (bar F)
patient #4; pRG-HCV IRES *wash* library	4280	5334	5071	5081	4925	4936	4161	4826,857	436,912	5G; 5J (bar G)
patient #7; pRG-HCV IRES *wash* library	863	789	829	880	823	928	821	847,571	46,267	5H; 5J (bar H)
patient #9; pRG-HCV IRES *wash* library	2485	2232	2667	2842	2398	2487	2415	2503,714	197,739	5I; 5J (bar I)

Figure S1 - Analysis of individual and collective activities of the HCV IRES in human CCL-13 cells using flow cytometry. Mean EGFP fluorescence per single cell was calculated from the population of DsRed2-positive CCL-13 cells. Each column represents seven independent experiments depicted in Table S2. * the most common sequence variant for patient #4.

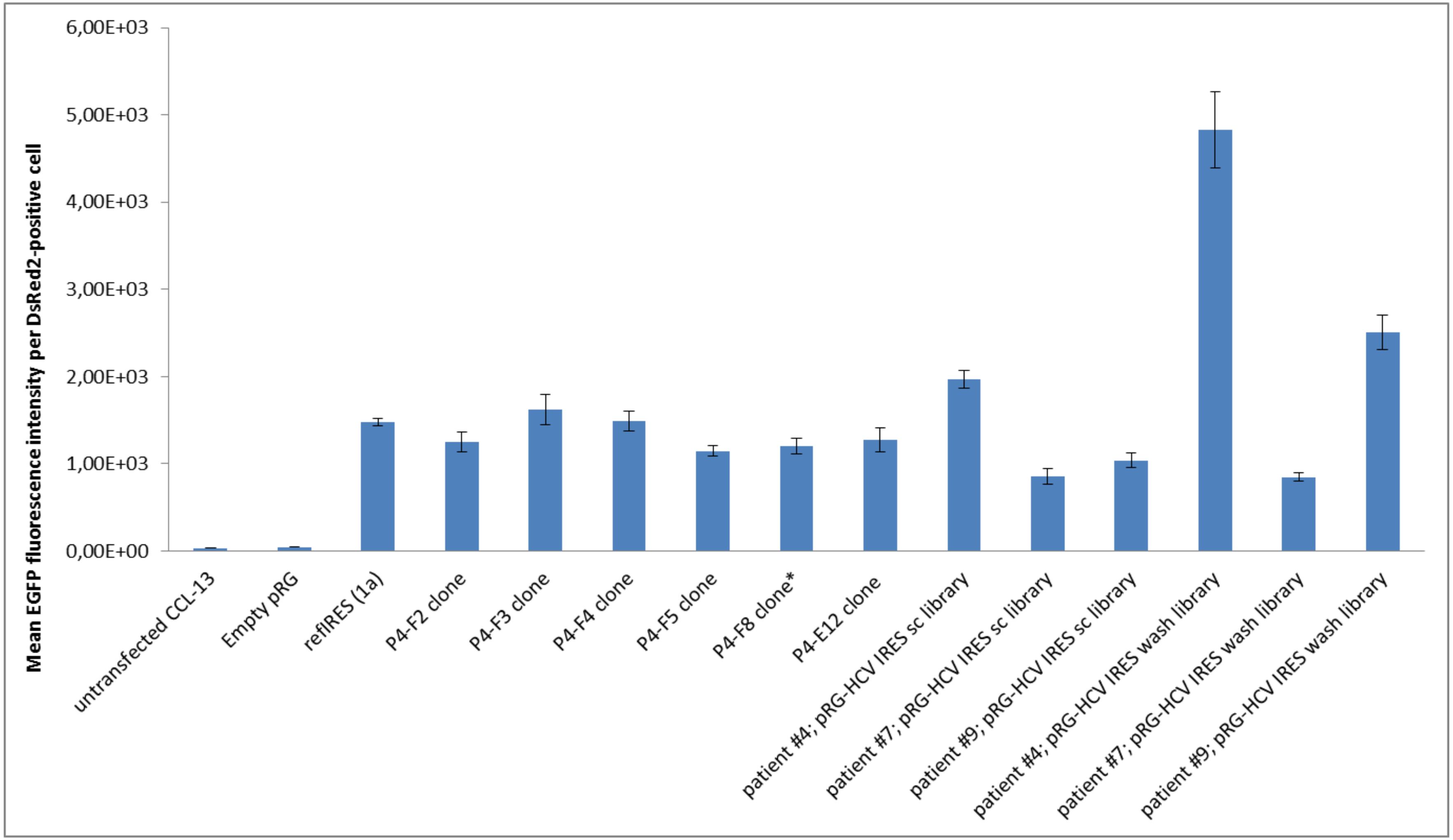

Supplement: Supplementary file 1 [file Data_Sheet_1.PDF]
